# Validation of a multiomic model of plasma extracellular vesicle PD-L1 and radiomics for prediction of response to immunotherapy in NSCLC

**DOI:** 10.1186/s13046-024-02997-x

**Published:** 2024-03-15

**Authors:** Diego de Miguel‑Perez, Murat Ak, Priyadarshini Mamindla, Alessandro Russo, Serafettin Zenkin, Nursima Ak, Vishal Peddagangireddy, Luis Lara‑Mejia, Muthukumar Gunasekaran, Andres F. Cardona, Aung Naing, Fred R. Hirsch, Oscar Arrieta, Rivka R. Colen, Christian Rolfo

**Affiliations:** 1 Center for Thoracic Oncology, Tisch Cancer Institute, Icahn School of Medicine at Mount Sinai, Mount Sinai, 1470 Madison Ave, New York, NY 10029 USA; 2 Marlene and Stewart Greenebaum Comprehensive Cancer Center, University of Maryland School of Medicine, Baltimore, MD USA; 3 University of Pittsburgh Medical Center, Pittsburgh, PA USA; 4 Hillman Cancer Center, University of Pittsburgh Medical Center, Pittsburgh, PA USA; 5 Medical Oncology Unit, A.O. Papardo & Department of Human Pathology, University of Messina, Messina, Italy; 6 Thoracic Oncology Unit, Instituto Nacional de Cancerología (INCan), Mexico City, Mexico; 7 Departments of Surgery and Pediatrics, Feinberg School of Medicine, Ann and Robert H. Lurie Children’s Hospital of Chicago, Northwestern University, Chicago, IL USA; 8 Molecular Oncology and Biology Systems Research Group (Fox G), Universidad El Bosque, Bogota, Colombia; 9 Departments of Investigational Cancer Therapeutics, The University of Texas MD Anderson Cancer Center, Houston, TX USA

**Keywords:** Non-small cell lung cancer, Immune-checkpoint inhibitors, Liquid biopsy, Biomarker, Extracellular vesicle PD-L1, Radiomics

## Abstract

**Background:**

Immune-checkpoint inhibitors (ICIs) have showed unprecedent efficacy in the treatment of patients with advanced non-small cell lung cancer (NSCLC). However, not all patients manifest clinical benefit due to the lack of reliable predictive biomarkers. We showed preliminary data on the predictive role of the combination of radiomics and plasma extracellular vesicle (EV) PD-L1 to predict durable response to ICIs.

**Main body:**

Here, we validated this model in a prospective cohort of patients receiving ICIs plus chemotherapy and compared it with patients undergoing chemotherapy alone. This multiparametric model showed high sensitivity and specificity at identifying non-responders to ICIs and outperformed tissue PD-L1, being directly correlated with tumor change.

**Short conclusion:**

These findings indicate that the combination of radiomics and EV PD-L1 dynamics is a minimally invasive and promising biomarker for the stratification of patients to receive ICIs.

**Supplementary Information:**

The online version contains supplementary material available at 10.1186/s13046-024-02997-x.

## Background

Immune-checkpoint inhibitors (ICIs) have showed unprecedented response rates in patients with non-small cell lung cancer (NSCLC), becoming the mainstay of treatment in patients without targetable mutations [1]. Nevertheless, the lack of biomarkers hinders the full benefit from this treatment. To date, only tissue PD-L1 and tumor mutational burden have been approved for guiding treatment selection in patients with NSCLC, however, they have showed suboptimal predictive performance without being able to explain the heterogeneity of outcomes [2, 3]. Indeed, tissue PD-L1, which is considered the standard-of-care biomarker for the prediction of tumor response to ICIs, has several limitations, including high variability between detection assays and complex spatial and temporal tumor heterogeneity, including changes observed after first-line treatments [4].

In this scenario characterized by the lack of biomarkers, our group has investigated novel predictive biomarkers combining the minimally invasive analysis of radiomics imaging and liquid biopsy [5]. Radiomics is the quantitative analysis of computed tomography (CT) or positron-emission tomography images to extract microscale quantitative data which has showed promising results at predicting response to immunotherapy [6–8]. Liquid biopsy measures biomarkers in body fluids, such as blood, allowing their longitudinal evaluation over the course of treatment. One of these biomarkers are extracellular vesicles (EVs), which are nanoparticles involved in cell signaling by the transference of cell cargo between different cells [9].

We showed that early dynamic expression of PD-L1 in extracellular vesicles (EVs) during treatment was able to predict durable response to ICIs as well as outcomes in a training and a validation cohort of patients with metastatic NSCLC. Moreover, we were able to combine the EV PD-L1 data with a radiomics model based on 6 radiomics features extracted from pre-treatment CT scan images in the training cohort of patients. This resulted in an improved specificity, sensitivity, and accuracy of the model to predict durable response [5]. Here, we aim to validate this multiparametric predictive model for treatment response in the validation cohort of patients.

## Main text

**Fig. 1:**
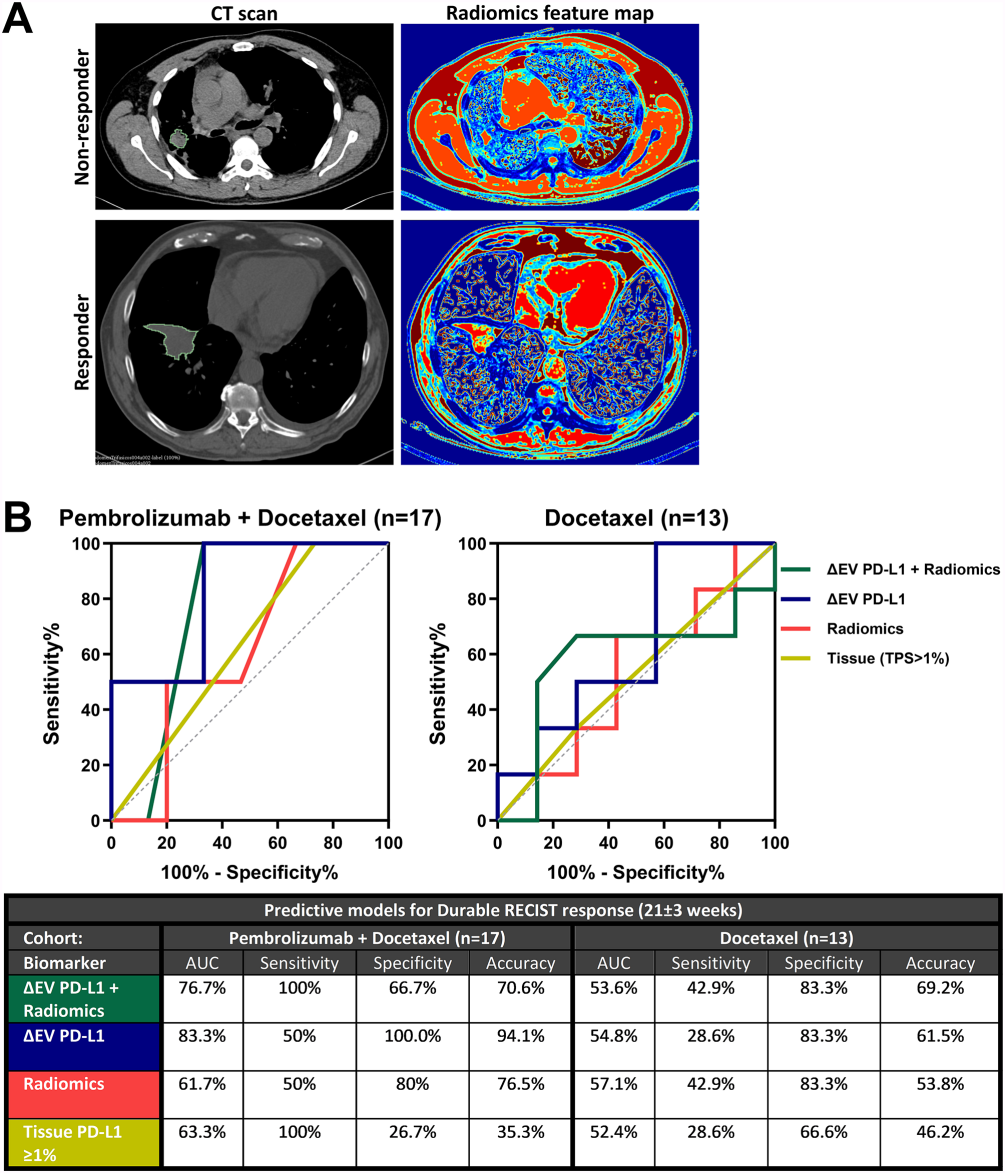
Predictive models for durable response: (**A**) Radiomic feature map sample of different expression of a radiomics feature (TL_F101: Range of Difference Variance) in non-responder and responder lesions from baseline computed tomography scans. (**B**) The predictive model of dynamics of ΔEV PD-L1 + Radiomics showed a 76.7% area-under-the-curve (AUC) to predict non-responders between patients receiving Pembrolizumab + Docetaxel, revealing an improvement in 15% over the Radiomics model and ~ 14% over the tissue PD-L1. To the contrary, only 56.3% AUC was found for ΔEV PD-L1 + Radiomics in the Docetaxel group, which was pretty similar to the one associated with tissue PD-L1 (binary logistic regression)

We performed the radiomics evaluation of pre-treatment CT scan images from our previously published model [5] in a validation cohort including 30 patients with advanced NSCLC who received second-line treatment with Pembrolizumab plus Docetaxel or Docetaxel alone from the phase 2 PROLUNG clinical trial [10]. Seventeen patients were treated with Pembrolizumab plus Docetaxel while 13 patients were treated with Docetaxel alone and used as comparative control (Supp. Table S1). Radiomics analysis of a total of 400 features in target and non-target lesions was performed according to our established methodology [5, 6]. Briefly, lesion segmentation was performed with the 3D Slicer 4.10.1 module including additional volumes of interest (VOI) of the normal pectoralis major muscle for within-phase normalization. For feature extraction, ten intensity-level histogram features and 195 Gy level co-occurrence matrix (GLCM) features were obtained. Then, we calculated 39 rotation-invariant texture features for each VOI and five gray levels. Moreover, 195 volume-dependent second-order features were calculated by dividing each GLCM feature by the volume of the segmented lesions. A representation of the values of this model is depicted in color in an example of a non-responder and a responder in Fig. 1A. We applied our trained model of 6 specific radiomic features (Supplementary table S2) into these 2 sets of patients and combined it with our previously described dynamic (Δ) EV PD-L1 analysis to predict durable response to ICIs (evaluated at 21 ± 3 weeks ~ 6 months).

As a result, the validation of the ΔEV PD-L1 plus radiomics revealed a 76.7% area-under-the-curve (AUC) at predicting response to Pembrolizumab plus Docetaxel at 6 months, outperforming tissue PD-L1 (Fig. 1B). This validates our previous results from the training cohort of patients, where the combined EV PD-L1 and radiomics showed similar AUC of 81.3% [5]. Moreover, it showed low AUC in patients with Docetaxel, which demonstrates the specificity of this biomarker at predicting response to ICIs (Fig. 1B). These results concur with those from a similar study which applied longitudinal deep-radiomics and clinical data for the prediction of durable response with 82.4% AUC. However, their model only showed a 58.8% AUC when considering baseline radiomics features and clinical data [11]. Other approaches combined radiomics with tissue PD-L1 RNA expression, but in fact showed only an AUC of 68% at predicting short-term response to ICIs at 3 months [12]. These lower performances could be related to the variety of treatment strategies included but also to the idea that dynamic and combined biomarkers tend to improve prediction over unique timepoint biomarkers [11, 13].

**Fig. 2:**
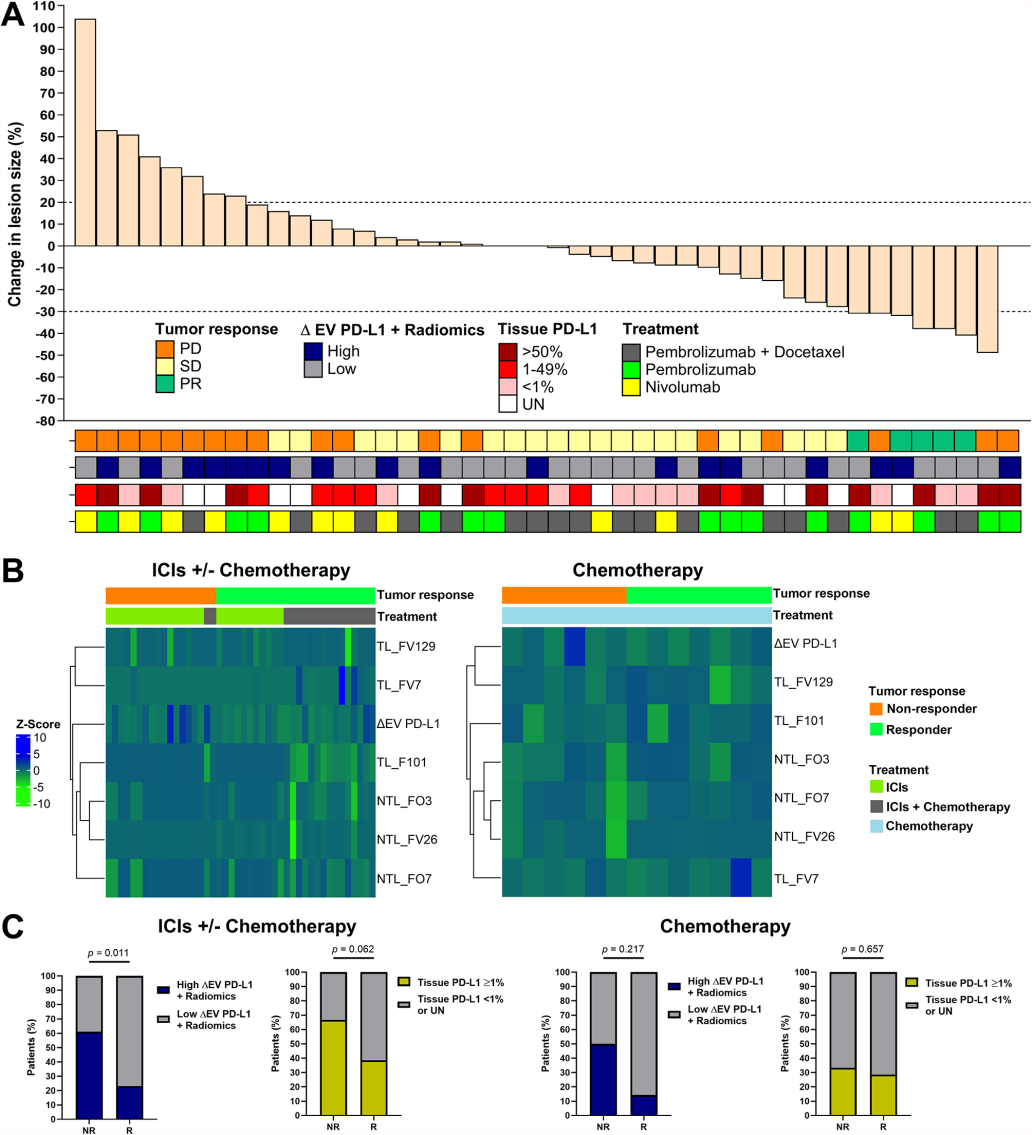
Durable tumor response to ICIs correlates with ΔEV PD-L1 + Radiomics. (**A**) Patients experiencing progressive disease (PD) (orange) showed higher ΔEV PD-L1 + radiomics values in comparison to those with stable disease (SD) and partial response (PR) (*p* = 0.008) (Kruskal–Wallis test) since those with high ΔEV PD-L1 + radiomics (blue) showed larger increases in tumor change (*p* = 0.047) (Mann–Whitney U test). Tissue PD-L1 tumor proportion score (TPS) was not associated with the tumor changes (*p* = 0.303) (*n* = 44). (**B**) Clustering heatmap of patient samples based on EV PD-L1 and radiomics features between responders and non-responders and according to treatment. (**C**) High ΔEV PD-L1 + radiomics predicted non-responders (*p* = 0.011) between patients receiving ICIs with or without chemotherapy while not in those receiving chemotherapy (*p* = 0.217). Tissue PD-L1 ≥ 1 was not associated with durable response to ICIs or chemotherapy (*p* = 0.062 & *p* = 0.657, respectively) (Chi-square tests)

Additionally, we included the training cohort of 27 patients to evaluate the performance of this combined predictive model in a more representative heterogeneous cohort of patients (Supp. Table S1). Thus, when all patients undergoing ICIs were analyzed (*n* = 44), ΔEV PD-L1 + radiomics levels were significantly associated with the type of response (*p* = 0.008) since those with high ΔEV PD-L1 + radiomics showed increasing tumor change (%) (*p* = 0.047) (Fig. 2A). To the contrary, tissue PD-L1 levels were not correlated (*p* = 0.303) and no association was observed between ΔEV PD-L1 + radiomics and response type or lesion size in the chemotherapy group. The representation of the clustered heat-map of radiomic features and ΔEV PD-L1 included in the predictive model for each patient can be observed in Fig. 2B. Variables were standardized using Z-score normalization (subtracting the mean of each variable and dividing by its standard deviation). “Euclidean” distance was used for cluster distance and “complete” for method using the ClusterHeatmap (version 2.14.0) and Circlize (version 0.4.15) libraries in R statistical software.

High ΔEV PD-L1 + radiomics identified non-responders (*p* = 0.011) showing 61.1% sensitivity and 76.9% specificity, while positive tissue PD-L1 (> 1%) was not associated to responders (*p* = 0.062). Indeed, responders showed lower expression levels of tissue PD-L1. In the group of patients receiving chemotherapy, none of these biomarkers were associated with the tumor response (Fig. 2C).

## Conclusions

Altogether, these results suggest that our multiparametric model based on EV PD-L1 dynamics and pre-treatment radiomics is a promising predictive biomarker to stratify patients with NSCLC to receive ICIs and could overcome the limitations of tissue PD-L1 testing. Despite preliminary, this is the first study to show validated evidence of the potential of the combined role of these biomarkers with specific role in ICIs. Nevertheless, we acknowledge the limitations of this work including a small sample size and the need for further validation in larger cohorts of patients.

### Electronic supplementary material

Below is the link to the electronic supplementary material.

Supplementary Material 1

## Data Availability

The datasets generated and/or analyzed during the current study are available from the corresponding author on reasonable request.

## Abbreviations

AUC: Area-under-the-curve
CT: Computed tomography
EV: Extracellular vesicle
GLCM: Gy level co-occurrence matrix
ICIs: Immune-checkpoint inhibitors
NSCLC: Non-small cell lung cancer
VOI: Volumes of interest
