# Supplementary material for: Validation of a multiomic model of plasma extracellular vesicle PD-L1 and radiomics for prediction of response to immunotherapy in NSCLC
Source: J Exp Clin Cancer Res. 2024 Mar 15;43:81. doi: 10.1186/s13046-024-02997-x (PMC10941547; doi:10.1186/s13046-024-02997-x)
Supplement: Supplementary file 1 — Supplementary Material 1 [file 13046_2024_2997_MOESM1_ESM.docx]

**Supplementary files for:**

**Commentary**

**Validation of a multiomic model of plasma extracellular vesicle PD-L1 and radiomics for prediction of response to immunotherapy in NSCLC**

Diego de Miguel‑Perez^1,2*^, Murat Ak^3,4*^, Priyadarshini Mamindla^4^, Alessandro Russo^2,5^,

Serafettin Zenkin^3^, Nursima Ak^3,4^, Vishal Peddagangireddy^3,4^, Luis Lara‑Mejia^6^, Muthukumar Gunasekaran^2,7^, Andres F. Cardona^8^, Aung Naing^9^, Fred R Hirsch^1^, Oscar Arrieta^6^, Rivka R. Colen^3,4^ and Christian Rolfo^1,2+^.

Supplementary table 1: Characteristics of the study populations.

|  | | **Pembrolizumab+Docetaxel (17)** | **Docetaxel (13)** | **ICIs (27)** |
| --- | --- | --- | --- | --- |
| **Characteristics** | | **Number of patients (%)** | **Number of patients (%)** | **Number of patients (%)** |
| **Gender** | Men | 9 (52.9%) | 3 (23.1%) | 18 (66.7%) |
|  | Women | 8 (47.1%) | 10 (76.9%) | 9 (33.3%) |
| **Age (years)** | Mean ± Standard deviation | 56.2 ± 14.6 | 62.9 ± 9.9 | 67.4 ± 11.7 |
| **Smoking habits** | Never smoker | 9 (52.9%) | 8 (61.5%) | 5 (18.5%) |
|  | Former smoker | 8 (47.1%) | 5 (38.5%) | 14 (51.9%) |
|  | Current smoker | 0 (0%) | 0 (0%) | 8 (29.6%) |
| **Histological subtype** | Non-SCC | 14 (82.4%) | 12 (92.3%) | 22 (81.5%) |
|  | SCC | 3 (17.6%) | 1 (7.7%) | 5 (18.5%) |
| **Stage** | IV | 15 (88.2%) | 10 (76.9%) | 26 (96.3%) |
|  | IIIB | 2 (11.8%) | 3 (23.1%) | 1 (3.7%) |
| **Immunotherapy treatment** | Pembrolizumab | 17 (100%) | 0 (0%) | 15 (55.6%) |
|  | Nivolumab | 0 (0%) | 0 (0%) | 12 (44.4%) |
| **Line** | First | 0 (0%) | 0 (0%) | 11 (40.7%) |
|  | Second | 17 (100%) | 13 (100%) | 14 (51.9%) |
|  | Third | 0 (0%) | 0 (0%) | 2 (7.4%) |
| **Tissue PD-L1 (TPS)** | Negative (<1%) | 6 (35.3%) | 3 (23.1%) | 5 (18.5%) |
|  | Low expression (1-49%) | 4 (23.5%) | 3 (23.1%) | 6 (22.2%) |
|  | High expression (≥50%) | 0 (0%) | 1 (7.7%) | 12 (44.4%) |
|  | Unknown | 7 (41.2%) | 6 (46.2%) | 4 (14.8%) |
| **Durable response** | Partial response | 2 (11.8%) | 1 (7.7%) | 3 (11.1%) |
|  | Stable disease | 13 (76.5%) | 6 (46.2%) | 8 (29.6%) |
|  | Progressive disease | 2 (11.8%) | 6 (46.2%) | 16 (59.3%) |
| **Progression** | Yes | 15 (88.2%) | 13 (100%) | 23 (85.2%) |
|  | No | 2 (11.8%) | 0 (0%) | 4 (14.8%) |
| **PFS (months)** | Median (range) | 12.2 (3.7-46.9) | 3.5 (1.8-14.0) | 5 (1.7-27.7) |
| **Death** | Yes | 11 (64.7%) | 10 (76.9%) | 15 (55.6%) |
|  | No | 6 (35.3%) | 3 (23.1%) | 12 (44.4%) |
| **OS (months)** | Median (range) | 18.4 (4.2-56.5) | 15.6 (3.9-46.7) | 12.4 (2.5-33.1) |

Supplementary table 2: Radiomics features included in the predictive model

| **Radiomic features** | | | |
| --- | --- | --- | --- |
| **Feature** | **Lesion Type** | **Level** | **Feature Name** |
| NTL_FV26 | Non-Target | 8 | Range of Information Measure Of Correlation 2 |
| NTL_FO3 | Non-Target | - | Mean of First order |
| TL_F101 | Target | 32 | Range of Difference Variance |
| TL_FV129 | Target | 64 | Average of Information Measure Of Correlation |
| TL_FV7 | Target | 8 | Average of Sum Variance |
| NTL_FO7 | Non-Target | - | 95% Percentile of First Order |
